# Extracorporeal Membrane Oxygenation in Congenital Diaphragmatic Hernia

**DOI:** 10.3389/fped.2019.00336

**Published:** 2019-08-08

**Authors:** Neysan Rafat, Thomas Schaible

**Affiliations:** Department of Neonatology, University Children's Hospital Mannheim, University of Heidelberg, Mannheim, Germany

**Keywords:** congenital diaphragmatic hernia, extracorporeal membrane oxygenation, pulmonary hypertension, surgical repair, long-term outcome

## Abstract

Congenital diaphragmatic hernia (CDH) is characterized by failure of diaphragmatic development with lung hypoplasia and persistent pulmonary hypertension of the newborn (PPHN). If conventional treatment with gentle ventilation and optimized vasoactive medication fails, extracorporeal membrane oxygenation (ECMO) may be considered. The benefits of ECMO in CDH are still controversial, since there are only few randomized trials demonstrating the advantages of this therapeutic option. At present, there is no precise prenatal and/or early postnatal prognostication parameter to predict reversibility of PPHN in CDH patients. Indications for initiating ECMO include either respiratory or circulatory parameters, which are also undergoing continuous refinement. Centers with higher case numbers and the availability of ECMO published promising survival rates, but data on long-term results, including morbidity and quality of life, are rare. Survival might be influenced by the timing of ECMO initiation and the timing of surgical repair. In this regard a trend toward early initiation of ECMO and early surgery on ECMO exists. The results concerning the cannulation modes are similar and a consensus on time limit for ECMO runs does not exist. The use of ECMO in CDH will continue to be evaluated, and prospective randomized trials and registry network are necessary to help answering the addressed questions of patient selection and management.

## Introduction

Congenital diaphragmatic hernia (CDH) is currently the most common indication for extracorporeal membrane oxygenation (ECMO) in neonates (1). Survival rates reported by the extracorporeal life support organization (ELSO) have continued to drop in the modern era (2) and systematic reviews concerning a benefit of ECMO in CDH did not find an advantage for ECMO (3–5). However, some centers and networks have demonstrated an increase in survival rates in CDH with the employment of ECMO by retrospective analysis in their series (6). ECMO may perform as a true safety net when conventional treatment strategies fail or may only be a marketing strategy for a center to become a high-volume center with the positive side effect of increasing experience in the treatment of CDH. By increasing experience in the treatment of CDH, ECMO employment might be reduced, however, in some cases of CDH, pulmonary hypertension is so severe that only ECMO support can provide a chance of survival. The pathophysiology of CDH includes lung hypoplasia and abnormal development of the pulmonary vasculature with hyper-reactivity which leads to persistent pulmonary hypertension of the newborn (PPHN) (1). Episodes of hypoxia and hypercapnia can exacerbate the PPHN, leading to severe morbidity and mortality. In patients who continue to have labile physiology and low preductal saturations despite optimal ventilation, inotropic and pulmonary vasodilatory support, the next intervention considered in the management of CDH is extracorporeal membrane oxygenation (ECMO), if available. We present a review of literature in this complex patient group and try to answer some questions about optimal time to start ECMO, recommended entry criteria, mode of ECMO, and timing of operation.

## Pathophysiology in CDH and Rationale for ECMO

**Figure 1:**
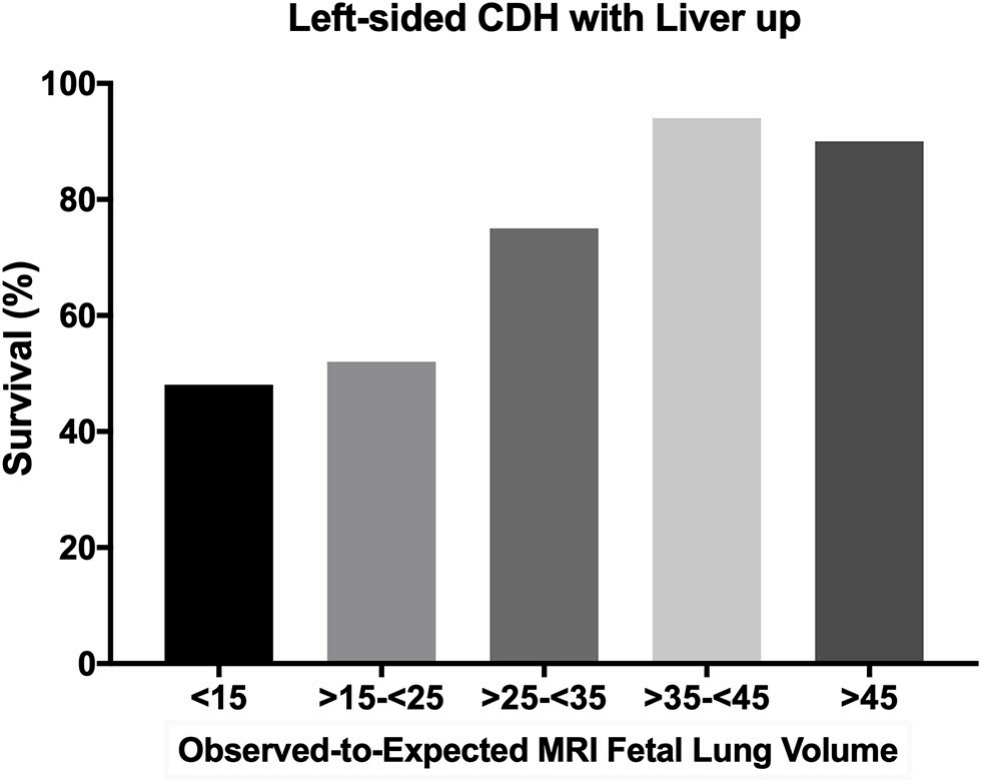
Survival rate of left sided CDH with liver up depending on the Observed-to-Expected MRI Fetal Lung Volume. All infants with left-sided CDH with liver up, treated in the years between 2001 and 2010 (*n* = 143) at our center in Mannheim, Germany were included for this analysis. This figure was newly created from previous published data (9). CDH, congenital diaphragmatic hernia.

In isolated CDH it seems possible to predict survival and need for ECMO and also chronic lung disease (CLD) by measuring the lung size by ultrasound or MRI. Liver herniation is also an independent risk factor for employment of ECMO (7). Data obtained by MRI seems to have lower interoperator variation and are easier to unify than data obtained by ultrasound. Categorization of severity was processed as previously described, and allows the comparison of results of different centers or ongoing studies investigating the effect of fetal tracheal occlusion (FETO) (8). As an example, we show our published data from 2006 concerning the prediction for ECMO in the group of left-sided CDH with liver up (Figure 1). Prognostic value in this group was only about 70%, because we could not predict how severe the pulmonary hypertension would be (9). With an optimal delivery room management, severely affected patients with liver up will present with some signs of a honeymoon (preductal saturation > 90%). That may be a reason for some optimism, since these patients with potential reversible pulmonary hypertension may benefit from ECMO. Avoiding stress, acidosis and severe hypoxemia and therefore crises of pulmonary hypertension reduces the need for ECMO. Surely some patients will be severely affected and do not have any sign of honeymoon, but an ongoing respiratory acidosis. In these cases, survival is impossible. But finding precisely the threshold for not offering ECMO is extremely difficult.

Using a multi-variate modeling to define the worst 10% of patients, Kays et al. included 172 consecutive inborn, prenatally diagnosed CDH patients in their analysis (10). Of the 19 worst patients, who were all aggressively resuscitated at birth and showed an average initial pH of 6.83 (at 1 h of life) with a PCO_2_ > 100, 10 of 16 patients, eligible for ECMO, survived to discharge (63%) (10). These results are difficult to compare to our data because the underlying prenatal severity is not published. In contrast, in our experience a preductal saturation <85% and/or arterial PCO_2_ > 100 after initial stabilization (at 1 h of life) is not compatible with survival despite early initiation of ECMO (within the first 4 h of life).

While evidence is missing for the necessity for employment of ECMO support, data from case series of ECMO centers offer convincing evidence of the potential for ECMO to rescue patients at the highly severe end of the CDH spectrum, a capability not well-documented for other treatment options besides ECMO so far (10). To understand the importance of ECMO support for selected cases of CDH, we will look at the pathophysiology of pulmonary hypertension in more detail.

Reduced lung volume available for gas exchange may lead to hypoxemia and hypercarbia, and is one of the main pathological abnormalities that can determine the indication for ECMO. One difficulty is that severe lung hypoplasia is not currently reversible in the short term, and can make the possibility of weaning from ECMO difficult or impossible. PPHN can arise even in minor lung hypoplasia by causing a right-to-left shunt and persistent fetal circulation, which further exacerbates hypoxemia and hypercarbia. The vasculature of small pulmonary arteries is pathologically thickened and reaches far into distal airways. Any kind of distress as barotrauma, inflammation or cytokine release by surgical intervention may induce additional vasoconstriction of the small vessels. The presence of PPHN is a significant predictor and cause of morbidity, need for ECMO and mortality in CDH (11). Antenatal markers which accurately predict the degree of PPHN in CDH, are not really known. To best address this challenge there are some suggestions, such as measuring antenatal pulmonary artery diameter, estimation of pulmonary perfusion by doppler measurements or left ventricular volume (12, 13).

Patients with either good or bad prognostication only based on antenatal lung volumes and ratios can have a discrepant clinical course. Postnatal clinical course could be a marker of severity of PPHN, with oxygenation index (OI) or combined parameters of ventilation and oxygenation (Wilford Hall/Santa Rosa Score) on day 1 predictive of outcome (14, 15). In postnatal therapy the institution of effective gentle ventilation from the very beginning in the delivery room, either by conventional ventilation or high frequency oscillatory ventilation (HFOV), is crucial. The VICI trial showed to some extent a benefit for conventional ventilation as the initial mode of ventilation in terms of a shorter ventilation time and a reduced need for ECMO, but not for mortality or BPD (16). The most widely used medication in PPHN management is inhaled nitric oxide (iNO). Inhaled nitric oxide (iNO) can improve oxygenation and reduce the acute need for ECMO in newborns with other causes of PPHN, but not conclusively in CDH (17). Nevertheless, the application of iNO in CDH is about 60% in reported case series (18).

Other treatment options like sildenafil or milrinone intravenously given in PPHN are applied to treat the condition in CDH with varying success (19). A European multi-center study is planned in order to investigate the effect of iNO vs. sildenafil after delivery. Systemic pressure should be maintained on normal values in order to avoid exacerbation of any right to left shunts.

**Figure 2:**
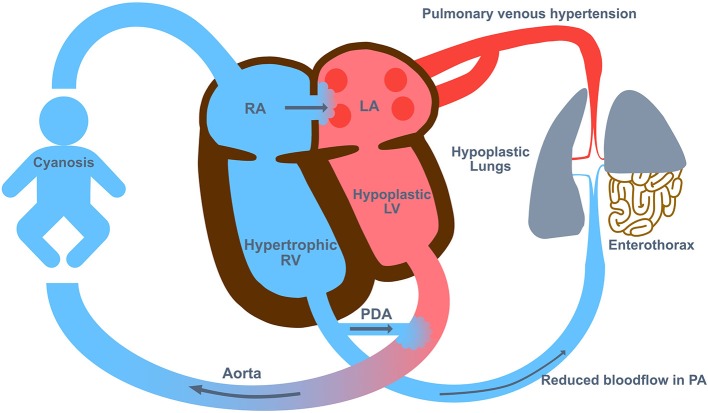
The Pathophysiology in congenital diaphragmatic hernia (CDH). The pathophysiology of CDH includes lung hypoplasia and abnormal development of the pulmonary vasculature with hyper-reactivity which leads to persistent pulmonary hypertension of the newborn (PPHN). LA, left atrium; LV, left ventricle; PA, pulmonary artery; PDA, persistent ductus arteriosus; RA, right atrium; RV, right ventricle.

Cardiac dysfunction resulting from the physiological derangements, PPHN or any associated congenital structural cardiac abnormality can complicate the clinical course (Figure 2). Poor cardiac output and impaired tissue oxygenation can ensue, and therefore serum lactate may be elevated and may work as an indication for employment of ECMO. In nearly all cases with suprasystemic pulmonary artery pressure and failure of reducing right-to-left shunts, a prostaglandin analog to open the ductus arteriosus and thereby unloading the right heart may help to stabilize the patient until ECMO is established.

Randomized controlled data on the role of ECMO in CDH is limited to two early ECMO studies and the UK ECMO trial (20–22). In the later trial, CDH patients were randomized at an oxygenation index of 40 to conventional ventilation vs. ECMO. From the 17 CDH patients randomized to conventional management all died, while in the ECMO group 4 of 18 infants survived (0 vs. 22% survival) (22). In 2006 a systematic review of ECMO in CDH was published, identifying 658 publications of which 21 (2,043 patients) met entry criteria (23). Looking at the findings of these studies, the authors concluded that employment of ECMO was associated with a reduction in CDH mortality (23). Zalla et al. (24) reviewed a single center CDH experience, dividing 16 years of treatment into four eras. In the latter two eras ECMO support was then available. *Post-hoc* analysis suggested a 73% reduction in risk of death in the ECMO eras compared to the pre-ECMO eras despite increases in CDH disease severity (24). In a recent analysis of the CDH EURO Consortium at four high volume centers in Europe, there are also higher survival rates reported from ECMO centers compared to non-ECMO centers (25).

There is only little evidence about the impact of the location of the hernia on ECMO need and postnatal prognosis. Patients with right-sided CDH have been identified as requiring increased use of ECMO [54% (26) and 71% (27)], but had better than expected ECMO survival [80% (26) and 83% (27)] (26, 27). Recently published data from the CDH study group are contrary to the before mentioned single center experiences (28). The survival without ECMO in left-sided CDH was higher compared to right-sided CDH and the use of ECMO was comparably low in right-sided CDH (36%) (28). The underlying pathophysiological mechanisms of these findings need to be investigated further. Some explanations for the different outcomes in right- vs. left-sided CDH have been suggested including the atypical dextroposition of the heart in left-sided CDH leading to adverse hemodynamic changes and an impaired cardiac function (27).

In general, improved survival without ECMO and also in ECMO centers is highly associated with the implementation of standardized treatment protocols, which mainly includes strategies to avoid ventilator associated lung injury (VALI) (29).

## CDH ELSO Indications vs. CDH EURO Consortium Indications

Entry criteria that accurately predict high mortality prior to the initiation of ECMO in infants with CDH have not been published (1). Various parameters have been used to predict those who benefit from ECMO (30–34), however, none of these criteria has been validated in multicenter studies (1). In the past, entry criteria suggested by ELSO for CDH patients included an oxygenation index (OI)>40 for 4 h or a PaO_2_ < 40 for 2 h (30), which were general entry criteria for ECMO for neonates with pulmonary hypertension of any cause. In the newest edition of their guidelines (1), ELSO has adopted their entry criteria for infants with CDH also according to the recommendations suggested by the CDH EURO Consortium Consensus −2015 Update (35). They define the following indications to initiate ECMO support in CDH patients: hypoxia, defined as preductal saturations consistently <80–85%; acidosis, defined as metabolic (lactate > 5 mmol/L or pH < 7.20) or respiratory (pH < 7.20 due to hypercarbia); hypercarbia, defined as persistent PaCO_2_ > 70 leading to pH < 7.20; or hypotension, defined as poor tissue perfusion, urine output <0.5 mL/kg/h or unresponsive to IV fluid and inotropic support. In addition, many centers use a specific limit on ventilator settings to avoid ventilator-associated lung injury (VALI) and transition to ECMO support when a patient does not respond appropriately (1). These include limiting the peak inspiratory pressure (PIP) (≤ 26 cm H_2_O, HFOV to a MAP of 14–15 cm H_2_O), and maintaining pH > 7.20 (usually PaCO_2_ < 70 mmHg) (1). The only minor differences in the recommendation of the CDH EURO Consortium Consensus relate to the hypercarbia leading to a pH < 7.15 and the ventilator settings (PIP > 28 cm H_2_O or MAP > 17 cm H_2_O) as an entry criteria (35). There are no significant differences between the two guidelines, and the combined entry criteria for ECMO in CDH allow a center-depend individual decision.

Relative contraindications to initiating ECMO support in CDH patients are comprised of significant congenital anomalies (major cardiac anomalies), lethal chromosomal abnormalities or other lethal malformations, Grade III/IV intracranial hemorrhage, prolonged mechanical ventilation requiring prolonged high pressure, weight <2 kg and gestational age < 34 weeks (1). The last two criteria are due to technical problems of vascular access and the complications of prematurity. In general, prematurity is more common in patients with congenital anomalies and accordingly also in CDH. Notably, preterm infants with CDH have an increased mortality compared to term infants (36).

## Timing of ECMO Deployment: Early vs. Late

The data of the CDH Study Group highlight the trend toward employing ECMO earlier (before CDH repair) as a component of preoperative stabilization (Red Book). While the inclusion criteria for initiating ECMO have been described in detail, there is no data available on the influence of the time point, when ECMO was initiated, on the morbidity and mortality in CDH patients. Many experts have the notion that the starting point of ECMO in CDH patients might be time-sensitive, since the responsivity of the pulmonary vasculature to pulmonary arterial hypertension treatment might be higher at the beginning and potentially reversible. If treated too late and complicated by the degree of pulmonary hypoplasia and VALI, the pulmonary hypertension can progress to right heart failure.

In our retrospective review of 321 neonates treated with ECMO from January 1987 to December 2006 at our center, we have already presented, that an early referral (<24 h) of CDH patients to the ECMO center correlated with an increased survival (6).

In a small cohort of patients with less than 15% predicted lung volume on antenatal scan who underwent EXIT to ECMO, no advantage in either survival or long-term morbidity could be demonstrated (37, 38). Although there are no randomized studies on EXIT to ECMO and these patients were not randomized, results led to the suggestion of little benefit for routine use (39).

## Type of Support: Veno-Venous vs. Veno-Arterial ECMO

The mode of ECMO in CDH, whether veno-venous (VV) or veno-arterial (VA), has not been demonstrated to affect survival so far, but the current available data is poorly controlled for underlying disease severity.

**Figure 3:**
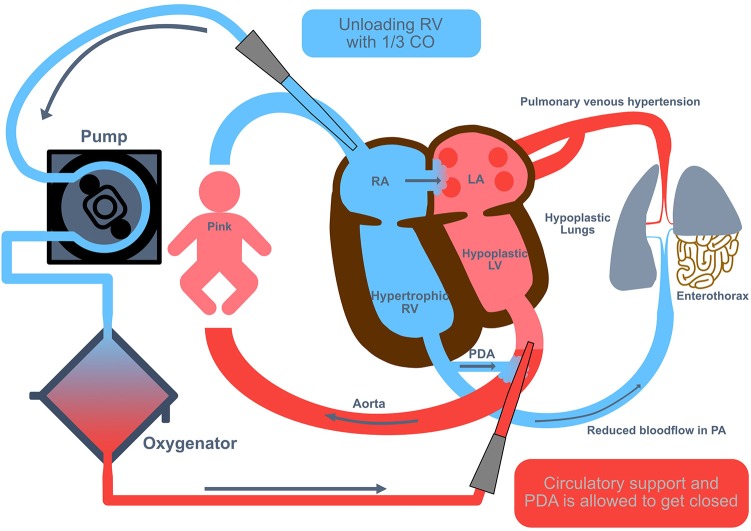
Veno-arterial extracorporeal membrane oxygenation in congenital diaphragmatic hernia (CDH). Veno-arterial (VA) extracorporeal membrane oxygenation (ECMO) is usually performed with an open cut-down technique where the right common carotid artery and right internal jugular vein are isolated and cannulated. Circuit flows are gradually increased to provide about 50 to 100 mL/kg/min, equivalent to an unloading of the right ventricle with 1/3 of the cardiac output. CO, cardiac output; LA, left atrium; LV, left ventricle; PA, pulmonary artery; PDA, persistent ductus arteriosus; RA, right atrium; RV, right ventricle.

Veno-arterial (VA) ECMO is usually performed with an open cut-down technique where the right common carotid artery (CCA) and right internal jugular vein (IJV) are isolated and cannulated. Circuit flows are gradually increased to provide about 50 to 100 mL/kg/min. Centrifugal ECMO circuits require smaller blood volumes for priming, but may be not as exact as roller-pump systems in providing low blood flows (the minimal possible blood flow without increased risk of clotting should be 30 mL/kg KG). Also, for appropriate hemodynamic reloading of the right ventricle in VA ECMO, especially in the weaning or the idling phase at the end of the ECMO support time, the Mannheim experience suggests to apply lower blood flows. The appropriate cannula size is determined depending on the infant's weight (sizes available down to 8 Fr; hence the VA technique may be feasible for the smaller infants). VA ECMO may have some advantages in infants with cardiac dysfunction (unloading of the right ventricle and maintaining good systemic output). Due to the pathophysiology with preexisting lower blood flow through the small pulmonary vascular bed in severely affected CDH patients, we always used VA ECMO in CDH patients in Mannheim (Figure 3).

It is often feasible to repair the CCA at decannulation although the rates of long-term patency are unclear (the IJV is usually ligated). Our own data of repair CCAs showed patency in half of the cohort, stenosis in about one fourth and occlusion in the remaining fourth part of the cohort (40).

**Figure 4:**
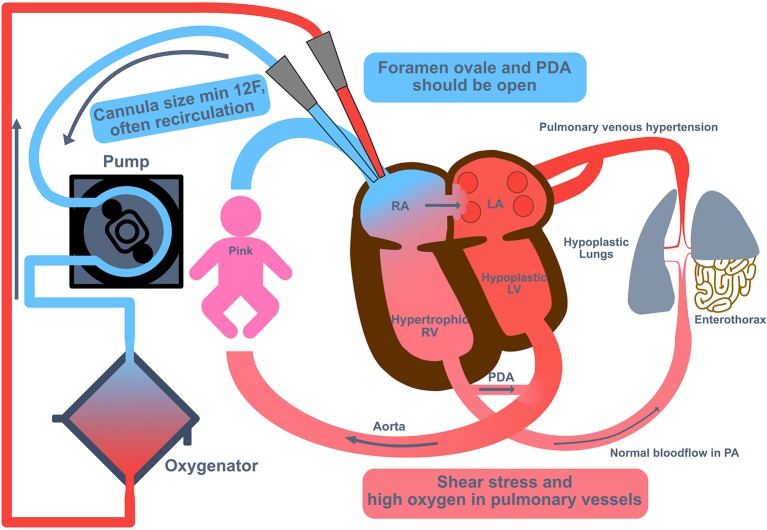
Veno-venous extracorporeal membrane oxygenation in congenital diaphragmatic hernia (CDH). Cannulation to Veno-venous (VV) extracorporeal membrane oxygenation (ECMO) can be performed by open surgery or using an ultrasound-guided percutaneous technique to cannulate the internal jugular vein (thereby preserving the common carotid artery). Because the cannulae are dual-lumen catheter, the smallest size used is 12 Fr, which requires the weight of the infant to be >2.5 kg. For unloading of the right heart, the duct should be open in the initial phase of VV ECMO. LA, left atrium; LV, left ventricle; PA, pulmonary artery; PDA, persistent ductus arteriosus; RA, right atrium; RV, right ventricle.

Cannulation to Veno-venous (VV) ECMO can be performed by open surgery or using an ultrasound-guided percutaneous technique to cannulate the IJV (thereby preserving the CCA). Because the cannulae are dual-lumen catheter, the smallest size used is 12 Fr, which requires the weight of the infant to be >2.5 kg. A potential advantage of this technique is that hyper-oxygenated blood is directed into the pulmonary artery and hence may reduce vascular resistance. But it is not clear whether a high amount of oxygen in the pulmonary vessels may lead to more inflammation via radical oxygen species (ROS). For unloading of the right heart, the duct should be open in the initial phase of VV- ECMO (Figure 4).

The VV-technique is dependent on satisfactory cardiac output and higher flows are usually required (often about 120 mL/kg/min). Recirculation of oxygenated blood up the venous lumen makes the precise catheter position more critical.

The cumulative ELSO experience on this topic was reviewed in two reports (41, 42), the latter covering 15 years from 1991 to 2006 (42). The report showed that VA ECMO was used in 82%, and VV ECMO only in 18% of the cases (42). Of the patients on VV ECMO 18% required conversion to VA ECMO, with survival dropping from 54% (VV) to 44% (switch from VV to VA), compared to 50% when ECMO was initiated as VA (42).

Although a systematic review suggested that there was no overall advantage with either the VV or VA technique, there is a difference in preferred mode of cannulation between centers (43). VA ECMO seems to be the more popular of the two modes, according to the scientific reports and ECMO registries; presumably as VA ECMO may give the additional benefit in the presence of severe cardiac dysfunction. They reported that VA was associated with slightly higher incidence of intracranial bleeding and seizure, while VV was associated with poorer renal perfusion. Size and vascular anatomy may sometimes dictate the mode used. Overall survival was similar between modes.

## Timing of Surgical Intervention: Before, During or After ECMO?

Typically, the duration of ECMO support in CDH patients takes between 1 and 4 weeks. It has been demonstrated that prolonged need for ECMO is associated with both increased morbidity and mortality (44). In our experience an optimal duration of our preferred ECMO mode (VA) in Mannheim is 7–14 days with an average duration of 10 days. Two days after successful weaning from ECMO support, surgical repair of the CDH will be performed. This traditional approach is supported by some studies, which identified an increased survival rate, if repair could be delayed until ECMO support has been completed (45, 46). Weaning off ECMO includes a trial to clamp off ECMO for 10–15 min, which is performed after 2 days with a very low flow of 30 ml/kg KG. The objective is achieving adequate oxygenation and ventilation (pCO_2_ < 60 mmHg) by a FiO_2_ ≤ 0.5 and gentle ventilation. In cases of weaning failure, we may prolong the ECMO support until a total of 21 days. If weaning fails after 21 days of ECMO support, we will offer compassionate/palliative care and a surgical repair will not be performed.

A main argument against surgical repair while on ECMO support is the higher incidence of bleeding. Complications from bleeding have, however, been reduced by careful anticoagulation management and the use of tranexamic acid perioperatively (47). Whether terminating treatment on ECMO without attempting surgical repair, might be a disadvantage and has not been systematically investigated. Some centers proclaim that surgical repair may increase the chances of survival, especially in the most severe forms of CDH. Yoder et al. reported from the CDH Study Group, that patients with a preductal saturation <85% in the first 24 h of life or before ECMO support, had an increased survival rate, if surgical repair was performed (44 vs. 23%) (48). Due to this finding, surgical repair while on ECMO support was advocated. Theoretically, surgical repair while on ECMO support may improve respiratory function by restoring normal anatomy, and intestinal complications from delaying surgery (ischemia or volvulus) are possible (49).

Analyzing the studies about the timing of surgical repair in more detail, the duration of ECMO support was shorter in patients with surgical repair after ECMO (8.4 days) compared to patients with surgical correction while on ECMO (8.9 days) (45). Also, patients undergoing surgery after ECMO support seemed to have lower severity of disease (45).

Another strategy to avoid mortality due to late or non-repair on ECMO is “early” repair on ECMO. In the study of Dassinger et al. 34 CDH patients underwent surgical repair while on ECMO at an average of 55 h after ECMO initiation (50). Only 9% of the patients suffered bleeding complications requiring intervention, and a total of 22 (71%) survived (50). The same trend toward an early surgical repair while on ECMO is supported by the data of Fallon et al. (51).

A last approach of a very early surgical repair in a honeymoon-like period before decompensation was suggested by Kays et al. (52). They developed a multi-variate modeling, employing anatomic and physiologic markers of severity—including prenatal lung measurements, liver position, birth physiology, and blood gas analysis data at 1 h after birth—to assess risk for ECMO at 1 h of life. Left-sided CDH patients with liver-up, which underwent surgical repair early before ECMO (mean time to surgery was 21 h), had a survival rate of 95%, compared to 65% in an equivalent group (left-sided CDH liver-up), who underwent ECMO support without previous surgical repair (52).

The optimal timing of surgery for patients on ECMO support is difficult to ultimately establish, but it seems that there is a developing consensus that repair at an earlier stage (within 1 week) with careful management of perioperative risks, may help with either weaning off ECMO or decisions on withdrawal later, and potentially improves outcome. It seems that different patients benefit from different strategies, therefore, we have to learn to individualize some aspects of CDH treatment, e.g., the timing of surgical repair.

## Improving Long-Term Outcome of CDH Via Structured Follow Up

The only outcome criteria of the ELSO-registered neonates with CDH are: transfer to another hospital or survival to discharge (53). In future, more attention should be directed to short and long-term morbidity. ECMO centers may reflect not only their survival rate, but also changes in indications for ECMO and cognitive impairment as the major long-term deficit following neonatal ECMO (54). The most important issue after ECMO in CDH is neurodevelopment outcome. During initial treatment, cerebral bleeding or infarction should be evaluated closely by cranial ultrasound. For long-term evaluation, MRI including angiography seems to be helpful for reflection of the initial decisions and may help to relate the results to the complications (40). Intelligence, memory, attention, behavior, and concentration deficits are domains of interest in school age (54–57).

Looking at the pulmonary outcome due to lung hypoplasia, the degree of CLD represents a short term complication (58); lung function testing may serve as a parameter for lung function development after severe neonatal disease (59). Furthermore, structured echocardiographic investigations may guide treatment and prognosis of pulmonary hypertension in CDH.

Surgical outcome parameters and complications after CDH repair include recurrence rates, feeding disorders and risk of ileus due to adhesions. In addition, further skeletal problems like chest wall deformations, funnel chest and scoliosis may occur. However, there is limited long-term outcome data in this regard.

Most follow-up studies in neonates and children who survived ECMO treatment have been cross-sectional, mono-center and in small study populations. With the current shift toward long-term multidisciplinary evaluations, observational follow-up-programs should be transferred toward risk stratification (60).

## Conclusion

In conclusion, the use of ECMO in CDH remains controversial. While many centers have demonstrated very good survival, utilizing minimal to no ECMO in CDH, the highest overall survival rates are reported by centers that employ ECMO, and whose patient populations likely also include the sickest patients. Due to the exceptional potential of ECMO to rescue CDH patients with profound pulmonary hypoplasia, the role of ECMO in severe CDH seems protected. The significance of surgical repair in attaining survival of patients with CDH, especially of those on the more severe end of the spectrum, cannot be overstated. While surgical repair after ECMO works well for those that successfully wean from ECMO, an early repair strategy on or before ECMO might potentially increase survival.

Based on the presented data and significant professional experience with CDH care, we feel confident that ECMO improves survival potential in more severe CDH compared to currently available non-ECMO techniques. Improving CDH survival is still a major goal and since the majority of deaths occur in those more severely affected, improving outcomes in those CDH patients treated with ECMO is essential. However, mortality should not be the only focus and parameter when proclaiming the importance of ECMO in CDH treatment. More research is needed to assess the morbidity of CDH patients after ECMO support and their long-term outcome.

In our opinion, factors which contribute to ongoing improvement, include having updated standardized postnatal treatment guidelines and participation in networks, further referrals to high-volume center with increasing experience in the treatment of CDH and severity-specific management.

## Author Contributions

NR and TS contributed equally to the concept and drafting of the article. Both authors approved the final version of the article.

### Conflict of Interest Statement

The authors declare that the research was conducted in the absence of any commercial or financial relationships that could be construed as a potential conflict of interest.
